# The metabolic effects of glucocorticoid replacement therapy in patients with secondary adrenal insufficiency due to hypothalamic-pituitary diseases: results from a retrospective and longitudinal study

**DOI:** 10.1007/s11102-026-01726-1

**Published:** 2026-07-11

**Authors:** Sabrina Chiloiro, Alessandra Vicari, Antonella Giampietro, Flavia Costanza, Rosalinda Calandrelli, Pier Paolo Mattogno, Quintino Giorgio D’Alessandris, Simona Gaudino, Laura De Marinis, Francesco Doglietto, Antonio Bianchi, Alfredo Pontecorvi

**Affiliations:** 1 Facoltà Di Medicina E Chirurgia, Università Cattolica Del Sacro Cuore, Rome, Italy; 2 Division of Endocrinology, Metabolic Disease and Internal Medicine, Fondazione Policlinico Universitario A. Gemelli IRCCS, Milan, Italy; 3 Dipartimento Di Scienze Radiologiche, Fondazione Policlinico Universitario A. Gemelli, Istituto Di Ricovero E Cura a Carattere Scientifico (IRCCS), Rome, Italy; 4 Dipartimento Di Neurochirurgia, Fondazione Policlinico Universitario A. Gemelli, Istituto Di Ricovero E Cura a Carattere Scientifico (IRCCS), Rome, Italy

**Keywords:** Central adrenal insufficiency, Secondary adrenal insufficiency, Pituitary disease, Hypothalamic disease, Glucocorticoid replacement therapy

## Abstract

**Background:**

Secondary adrenal insufficiency (SAI) is a complex endocrine disorder. Glucocorticoid (GC) replacement therapy is crucial for ensuring patient survival and guaranteeing an adequate quality of life. GC replacement therapy requires a balance between undertreatment, with the consequent risk of adrenal crisis, and overtreatment, which can have long-term effects on the metabolic and cardiovascular systems.

**Methods:**

We conducted a retrospective, longitudinal, observational study on 140 patients affected by pituitary disease with at least 3 years of follow-up. Patients were consecutively included in the study with a ratio 1:1, considering patients affected by SAI and who were therefore on GCs replacement therapy, and patients with pituitary disease without SAI (controls).

**Results:**

Worsening of glucose metabolism occurred in 29 patients with SAI (64.4%) and in 16 controls (35.6%, *p* = 0.019). Worsening of lipid metabolism occurred in 52 patients with SAI (56.5%) and in 40 controls (43.5% *p* = 0.033). GC replacement therapy (*p* = 0.014, OR: 2.3, 95%IC: 1.1–4.9), higher fasting glycaemia at baseline (*p* = 0.04, OR: 7.6, 95%IC: 2.3–25.4), IGT/T2DM at baseline (*p* = 0.016, OR: 8.6, 95%IC: 0.8–57) were the main risk factors for the worsening of glucose metabolism at 3-year follow-up. TSH deficit remained the only risk factor for the worsening of lipid profile (*p* < 0.001, OR: 2.9, 95%IC: 1.3–6.5).

**Conclusion:**

Our study proved that GCs replacement therapy may be associated with a worsening of glucose metabolism, particularly in patients already affected by IGT/T2DM. Tailored and holistic management is essential for the management of GCs replacement therapy, in patients with other metabolic disorders and pituitary hormone deficits.

## Introduction

Secondary adrenal insufficiency (SAI) is a relatively uncommon endocrine disorder with an estimated prevalence of 150–280 cases per million people, and an increased risk of mortality [1]. SAI is due to an impaired secretion of glucocorticoids from the adrenal cortex, due to a lack of stimulation by the pituitary gland’s adrenocorticotropic hormone (ACTH) [2]. Glucocorticoid (GC) replacement therapy is mandatory in patients with SAI, as it constitutes a life-saving intervention [3, 4]. Several formulations of glucocorticoids are available nowadays: the most used are short-acting glucocorticoids, such as cortisone acetate and hydrocortisone, and sustained-release formulations [5–7]. SAI represents a significant challenge in the modern neuroendocrinology, as numerous constraints exist regarding the dosage and monitoring of GC replacement therapy [3, 8]. In the absence of biochemical markers, the dosage of GC in hormone replacement therapy is primarily determined by the patient’s clinical condition, aiming to mimic the physiological circadian rhythm of cortisol secretion [9]. The main issue in the management of patients with SAI is the optimization of dosages of GC for replacement therapy to improve symptoms of SAI, to reduce the risk of adrenal crises and the risk of potential overtreatment [10–14].

GC replacement therapy has been shown to impact on cardiovascular function, glucose, lipid and bone metabolisms, mood alterations and immune disorders [13, 15–17]. In the study by Stewart et al., the odds ratios compared with matched controls were as follows: 1.87 for diabetes mellitus, 1.98 for hyperlipidemia, 2.24 for hypertension, 2.55 for depression, and 2.80 for anxiety [18]. Filipsson et al. demonstrated that patients undergoing GC replacement therapy had higher waist circumference (*p* < 0.01), waist-hip ratio *p* < 0.01), glycosylated hemoglobin (HbA1C) (*p* < 0.0001), total cholesterol (*p* < 0.05), and triglyceride (*p* < 0.001) levels [19].

Therefore, in this study we investigated the effects of GC replacement therapy on cardiovascular system and on glucose and lipid metabolisms in patients with SAI.

## Patients and methods

We conducted a retrospective, longitudinal, monocentric, observational, cross-sectional study at the Pituitary Unit of the Department of Endocrinology and Metabolic Diseases of Gemelli University Hospital in Rome.

### Objectives

The primary objective of this study was to compare the outcome of glucose metabolism between patients with SAI on GC replacement therapy and patients without SAI.

The secondary objective was to compare the outcomes of lipid metabolism between the two groups of patients.

### Inclusion and exclusion criteria

Patients were consecutively enrolled in the study according to the following inclusion/exclusion criteria.

Inclusion criteria were:

- patients with history of pituitary mass, such as secreting and non-secreting pituitary adenoma/neuroendocrine tumors (PAs/PitNETs);
- patients who were considered cured for their primary pituitary disease after surgery and remained in remission during the time of observation;
- patients with at least three years of observation/follow-up;
- patients with available data of metabolic parameters (fasting glucose, HbA1C, total cholesterol, high density lipoprotein—HDL—cholesterol, calculated low density lipoprotein -cLDL—cholesterol, triglycerides) at baseline and at follow-up;
- patients with stable dose for all hormone replacement therapies during the three years of observation/follow-up;
- patients with age ≥ 18 years;
- patients who agree to participate to the study.

Exclusion criteria were:

- history of bariatric surgery;
- concomitant therapies impacting glucose and lipid metabolism and cardiovascular function;
- corticosteroid therapy for other diseases;
- patients with metabolic parameters (glucose, HbA1C, total cholesterol, HDL, cLDL, triglycerides) not conducted in fasting status, that was verified during clinical evaluation, according to clinical practice;
- patients undergoing GLP-1RA therapy for weight loss and gliflozin therapy;
- patients who needed adjustments of dose of hormone replacement therapy and/or prescription of new hormone replacement therapies.

### Study protocol

#### Data collection

For each patient included in the study, the following data were collected from medical records:

- at baseline (first endocrinological assessment prior to surgery): gender; age at diagnosis; primary pituitary disease; pituitary hormone deficits; presence of prediabetes/diabetes and dyslipidaemia, fasting glucose levels, HbA1C, total cholesterol, HDL, cLDL, triglycerides, body mass index (BMI), and concomitant therapies.
- at 3 years observation: pituitary hormone deficits; presence of prediabetes/diabetes and dyslipidaemia, fasting glucose levels, HbA1C, total cholesterol, HDL, cLDL, triglycerides, BMI, concomitant therapies and hydrocortisone or equivalent daily dose.

To determine the glucocorticoid equivalent, the total daily dose of cortisone acetate was converted to hydrocortisone equivalents using a standard equivalence factor of 0.8 (corresponding to the therapeutic ratio of 25 mg of cortisone acetate to 20 mg of hydrocortisone).

#### Patients and controls

Patients were consecutively included in the study with a ratio 1:1, considering patients affected by SAI and who were therefore on GCs replacement therapy, and patients with pituitary disease in the absence of SAI (control group).

### Diagnosis of SAI in patients under investigation

SAI was diagnosed according to last 2016 Endocrine society practice guidelines [4]. Cortisol levels between 8 and 9 a.m. (at least 18 h after the last hydrocortisone dose) was applied as a first-line diagnostic test. SAI was therefore diagnosed if cortisol levels were below 3 µg/dL. For patients with morning cortisol levels between 3 and 15 µg/dL, we performed a corticotropin stimulation test. In this case, a peak cortisol levels below 18.1 µg/dL after 30 and 60 min indicated adrenal insufficiency.

### Diagnosis of other hormonal deficiencies in patients under investigation

According to recent guidelines, hypogonadal hypogonadism was defined as low circulating testosterone (≤ 12 nmol/L), low/inappropriately normal pituitary gonadotropins, and relevant symptoms [20]. The diagnosis of thyroid-stimulating hormone (TSH) deficiency was made by biochemical testing in individuals with low circulating free thyroxine (FT4) and low or normal serum TSH [21]. Growth hormone deficiency (GHD) was diagnosed, according to recent guidelines, in patients with panhypopituitarism and a low IGF-I, or in patients with pathological stimulation tests [22, 23]. Arginine vasopressin deficiency (AVPD) was defined by polyuria (urine output of more than 50 ml/kg/24 h) and polydipsia, and a positive result of the water deprivation rtest, or hypertonic saline test plus plasma copeptin measurement [24].

### Evaluation of glucose metabolism

To evaluate glucose metabolism, the following data were collected: fasting glycaemia, HbA1C, diagnosis of impaired fasting glucose (IFG), or impaired glucose tolerance (IGT) or type 2 diabetes mellitus (T2DM), treatment with hypoglycaemic drugs. IFG was defined as a fasting plasma glucose value ranging from 100 mg/dL to 125 mg/dL [25], IGT was defined as a 2-h plasma glucose value during a 75 g OGTT ranging from 140 mg/dL to 199 mg/dL [25], and T2DM diagnosed based on either two occurrences of an HbA1C > 6.5% (48 mmol/mol), fasting glycemia > 126 mg/dL, or a 2-h plasma glucose value during a 75 g oral glucose tolerance test (OGTT) ≥ 200 mg/dL [25].

At follow-up, the glucometabolic status was defined:

- improved, in case of improvement of serum glucose parameters and/or reduction/withdrawal of anti-diabetic treatments;
- worsened, in case of worsening of serum glucose parameters and/or increased dosage or new prescription of anti-diabetic treatments;
- unchanged, in case not clinically significant variations of glucose parameters and/or maintenance of the same anti-diabetic treatments.

The decision to start medication for glucose metabolism abnormalities was based on deterioration in laboratory parameters, following an initial biochemical test evaluation that did not indicate the need for specific drugs.

The changes in glucose/HbA1c parameters were considered clinically significant if there was a transition from normal fasting glucose or normal glucose tolerance to impaired fasting glucose (IFG) or impaired glucose tolerance (IGT), or from IFG/IGT to type 2 diabetes mellitus (T2DM), according to the diagnostic criteria [25].

### Evaluation of lipid metabolism

To evaluate lipid metabolism, the following data were collected: total cholesterol, HDL, cLDL, cholesterol, triglycerides, treatment with lipid-lowering drugs. Hypercholesterolemia was defined as a plasma concentration of total cholesterol > 200 mg/dL or cLDL ≥ 130 mg/dL [26]; hypertriglyceridemia was defined as triglycerides ≥ 150 mg/dL [26].

At follow-up, the cholesterol status was defined:

- improved, in case of reduction/withdrawal of lipid-lowering drugs and/or improvement in serum lipid parameters;
- worsened, in case of increased dosage or new prescription of lipid-lowering drugs or worsening of serum lipid parameters;
- unchanged, in case of maintenance of the same lipid-lowering drugs or in case of not clinically significant variations of lipid parameters.

The decision to start medication for lipid metabolism abnormalities was based on deterioration in laboratory parameters, following an initial biochemical test evaluation that did not indicate the need for specific drugs.

The changes in lipid parameters were considered clinically significant if there was a transition from one LDL cholesterol or triglyceride risk class to another, according to the diagnostic criteria [26, 27].

### Statistical analysis

Descriptive statistics were used to report on the clinical and demographic characteristics of the patient cohort. The normality of continuous variables was tested using the Kolmogorov–Smirnov test. Quantitative variables were expressed as median and interquartile range (IQR) and qualitative variables as absolute and percentage frequencies. All variables were subjected to univariable logistic analysis, and covariates found to be associated (exploratory univariate *p* < 0.05) with rejection were entered into a multivariable logistic regression model as independent covariates. A stepwise selection method (*p* < 0.05) and the Ridge regression were applied to identify the final regression model. Analyses were performed with SPSS software version 24.0 for Windows.

## Results

### Baseline assessment

A total of 140 patients were included in the study: 70 patients affected by SAI and on treatment with GC replacement therapy, and 70 patients with pituitary disease in the absence of SAI (control group).

Among the whole study population, seventy-two patients were male (51.4%). The median age at baseline was 44 (IQR: 25). The study cohort consisted of 81 patients with non-secreting PAs/PitNETs (57.9%), 29 patients with GH-secreting PAs/PitNETs (20.7%), 6 patients with mixed GH/PRL-secreting PAs/PitNETs (4.3%), 18 patients with PRL-secreting PAs/PitNETs (12.9%) and 6 patients with ACTH-secreting PAs/PitNETs (4.3%). All patients were considered cured after surgery and remained controlled during the three years of observation. At baseline, 47 patients (33.6%) were affected also by hypogonadal hypogonadism, 50 patients by TSH deficit (35.7%), 29 patients by GHD (20.7%), 14 patients by AVP-D, 21 patients by primary hypothyroidism (15%). Among patients with hypogonadal hypogonadism, 30 were undergoing replacement therapy. Among patients with GHD, 14 were undergoing recombinant human growth hormone (rhGH) replacement therapy. Twelve patients were affected by IGT, IFG or T2DM (8.6%) and 25 patients were affected by dyslipidemia (17.9%).

Patients with SAIs were more frequently males (58.3%) than controls (41.7%, *p* = 0.04). Age and primary pituitary disease did not differ among patients with SAI and controls, as reported in Table 1. Median daily dose of hydrocortisone or equivalent was 25 mg (IQR: 15 mg, range 10–55 mg). Patients with SAIs were more frequently affected by hypogonadal hypogonadism (78.8%) than controls (21.3% *p* < 0.001), by TSH deficit (80%) than controls (20% *p* < 0.001), by GH deficit (79.3%) than controls (20.7% *p* < 0.001), by arginine-vasopressin (AVP) deficit (100%) than controls (0% *p* < 0.001), as reported in Table 1.

**Table 1:** Clinical and biochemical features of study population before starting GCs replacement therapy. Univariate analysis

	Patients with SAI on GCs replacement therapy	Controls	*p*-value
Gender			0.04
Males n, (%)	42 (58.3%)	30 (41.7%)	
Females n, (%)	28 (41.2%)	40 (58.8%)	
Age median, years (IQR)	45 (28)	59 (27)	0.653
Primary pituitary diseases			0.346
Not-secreting lesions n, (%)	44 (54.3%)	37 (45.7%)	
Cushing disease n, (%)	4 (66.7%)	2 (33.3%)	
Acromegaly n, (%)	14 (40%)	21 (60%)	
Hyperprolactinemia n, (%)	8 (44.4%)	10 (55.6%)	
Hypogonadal hypogonadism			0.001
Yes, n (%)	37 (78.7%)	10 (21.3%)	
No, n (%)	33 (35.5%)	60 (64.5%)	
TSH deficit			0.001
Yes, n (%)	40 (80%)	10 (20%)	
No, n (%)	30 (33.3%)	60 (66.7%)	
GHD			0.001
Yes, n (%)	23 (79.3%)	6 (20.7%)	
No, n (%)	47 (42.3%)	64 (57.7%)	
AVP deficit			0.001
Yes, n (%)	14 (100%)	0 (0%)	
No, n (%)	56 (44.4%)	70 (55.6%)	
Glucose metabolism at baseline			0.998
Normal, n (%)	64 (50%)	64 (50%)	
IGT/DM2, n (%)	5 (50%)	5 (50%)	
Dyslipidemia at baseline			0.508
No, n (%)	56 (48.7%)	59 (51.3%)	
Yes, n (%)	14 (56%)	11 (44%)	

Frequency of IGT, IFG, T2DM and dyslipidemia did not differ among patients with SAI and controls (respectively *p* = 0.998 and *p* = 0.508, as reported in Tables 1 and 2). Among patients with SAI, five patients were affected by IGT, IFG or T2DM (5/70, 7.1%) and 14 patients were affected by dyslipidemia (20%). Among controls, five patients were affected by IGT, IFG or T2DM (5/70, 7.1%) and 11 patients were affected by dyslipidemia (15.7%). Fasting glycemia, HbA1C, HDL-cholesterol, cLDL, triglycerides did not significantly differ among patients with SAI and controls, as reported in Table 1.

**Table 2:** Glucose and lipid metabolism before and after 3 years of observation. Univariate analysis

	Patients with SAI on GCs replacement therapy	Controls	*p*-value
Glucose metabolism at baseline			
Normal, n (%)	64 (50%)	64 (50%)	0.998
IGT/DM2, n (%)	6 (50%)	6 (50%)	
Fasting glycemia mg/dL at baseline median (IQR)	102 (12)	92 (21)	0.913
Glycosylated hemoglobin at baseline mol/mol (IQR)	48 (25)	42 (5)	0.413
Dyslipidemia at baseline			
No, n (%)	56 (48.7%)	59 (51.3%)	0.508
Yes, n (%)	14 (56%)	11 (44%)	
HDL-cholesterol (mg/dL) median (IQR)	52 (24)	65 (30)	0.81
LDL-cholesterol (mg/dL) median (IQR)	118 (48)	92 (58)	0.947
Triglycerides (mg/dL) median (IQR)	112 (80)	100 (132)	0.756
BMI at baseline Kg/m^2^ median (IQR)	25.8 (7)	28 (9)	0.48
Glucose metabolism at FUP			
Normal	41 (43.2%)	54 (56.8%)	0.019
IGT/DM2	29 (64.4%)	16 (35.6%)	
Fasting glycemia mg/dL at FUP median (IQR)	91 (29)	99 (33)	0.02
Glycosylated hemoglobin mol/mol at FUP (IQR)	44 (8)	40 (11)	0.255
Dyslipidemia at FUP			
No	18 (37.5%)	30 (62.5%)	0.033
Yes	52 (56.5%)	40 (43.5%)	
HDL-cholesterol mg/dL at FUP median (IQR)	56 (19)	47 (19)	0.037
LDL-cholesterol mg/dL at FUP median (IQR)	120 (57)	113 (47)	0.78
Triglycerides mg/dL at FUP median (IQR)	125 (85)	100 (66)	0.02
BMI at FUP Kg/m^2^ median (IQR)	30 (9)	29 (9)	0.97

### Follow-up data

#### Follow up data – changes in glucose metabolism for patients with SAI and controls

After 3 years of follow-up, glucose metabolism worsened in 45 patients with the development of IGT/T2DM (32.1%). Patients suffering from SAI and therefore treated with GC replacement therapy had a higher incidence of glucose metabolism alterations (64.4%, *p* = 0.019, OR: 2.39, 95% IC: 1.15–4.97) than controls (respectively 35.6%, OR: 0.67, 95%IC: 0.49–0.92). Among the whole study population, the worsening of glucose metabolism occurred more frequently in patients with previous history of acromegaly (62.1%, *p* = 0.003), in patients affected by IGT/T2DM at baseline (91.7%, *p* < 0.001), in patients with higher fasting glucose (*p* < 0.001) and higher HbA1C (*p* = 0.024) levels at baseline. The worsening of glucose metabolism did not correlate with gender, age, concomitant hypogonadal hypogonadism, TSH deficit, GHD, AVP deficit, types of glucocorticoids (cortisone acetate or hydrocortisone) and daily dose of hydrocortisone or equivalent, as reported in Table 3.

**Table 3:** Glucose metabolism outcome at 3 years of GCs replacement therapy in patients with SAI and at 3 years of observation in controls, according to different clinical and biochemical features. Univariate analysis

		Glucose metabolism at FUP		
		Normal/unchanged	Worsening	*p*-value
Number of patients, (%)		95 (67.9%)	45 (32.1%)	n.a
Gender				0.428
Males n, (%)		51 (70.8%)	21 (29.2%)	
Females n, (%)		44 (64.7%)	24 (35.3%)	
Age median, years (IQR)		36 (35)	49.5 (29)	0.246
Primary pituitary diseases				0.003
Not-secreting lesions n, (%)		61 (75.3%)	20 (24.7%)	
Cushing disease n, (%)		4 (66.7%)	2 (33.3%)	
Acromegaly n, (%)		15 (42.8%)	20 (57.2%)	
Hyperprolactinemia n, (%)		15 (83.3%)	3 (16.7%)	
Hypogonadal hypogonadism				0.732
Yes, n (%)		31 (66%)	16 (34%)	
No, n (%)		64 (68.8%)	29 (31.2%)	
Therapy for hypogonadal hypogonadism				0.549
Yes, n (%)		19 (63.3%)	11 (36.7%)	
No, n (%)		76 (69.1%)	34 (30.9%)	
Secondary TSH deficit				0.686
Yes, n (%)		35 (70%)	15 (30%)	
No, n (%)		60 (66.7%)	30 (33.3%)	
Therapy for TSH deficit (levothyroxine)				0.586
Yes, n (%)		50 (65.8%)	26 (34.2%)	
No, n (%)		45 (70.3%)	19 (29.7%)	
GHD				0.555
Yes, n (%)		21 (72.4%)	8 (27.6%)	
No, n (%)		74 (66.7%)	37 (33.3%)	
Hormone replacement therapy with rhGH				0.132
Yes, n (%)		12 (85.7%)	2 (14.3%)	
No, n (%)		83 (65.9%)	43 (34.1%)	
AVP deficit				0.763
Yes, n (%)		9 (64.3%)	5 (35.7%)	
No, n (%)		86 (68.3%)	40 (31.7%)	
Glucocorticoid type				0.927
Hydrocortisone n, (%)		28 (58.3%)	20 (41.7%)	
Cortisone Acetate n, (%)		12 (57.1%)	9 (42.9%)	
Hydrocortisone or equivalent dose mg/day median (IQR)		30 (19)	20 (15)	0.509
HCEq/BSA		14 (8.5)	9.7 (8.6)	0.062
Hydrocortisone dose or equivalent	< 20 mg daily	12 (29.3%)	13 (44.8%)	0.29
	20–29 mg daily	7 (17.1%)	5 (17.2%)	
	> 30 mg daily	22 (53.7%)	11 (37.9%)	
Fasting glycemia mg/dL at baseline median (IQR)		95 (5)	102 (19)	0.001
Glycosylated hemoglobin at baseline mol/mol (IQR)		41 (10)	48 (18)	0.024
Glucose metabolism at baseline				0.001
Normal n, (%)		94 (73.4%)	34 (26.6%)	
IGT/DM2 n, (%)		1 (8.3%)	11 (91.7%)	
Dyslipidemia at baseline				0.061
No n, (%)		82 (71.3%)	33 (28.7%)	
Yes n, (%)		13 (52%)	12 (48%)	
Glucose metabolism treatment				0.001
None n, (%)		95 (100%)	0 (0%)	
Diet n, (%)		0 (0%)	13 (100%)	
Oral hypoglycemic drugs, n (%)		0 (0%)	24 (100%)	
Insulin, n (%)		0 (0%)	8 (100%)	

#### Follow up data – changes in lipid metabolism for patients with SAI and controls

**Fig. 1:**
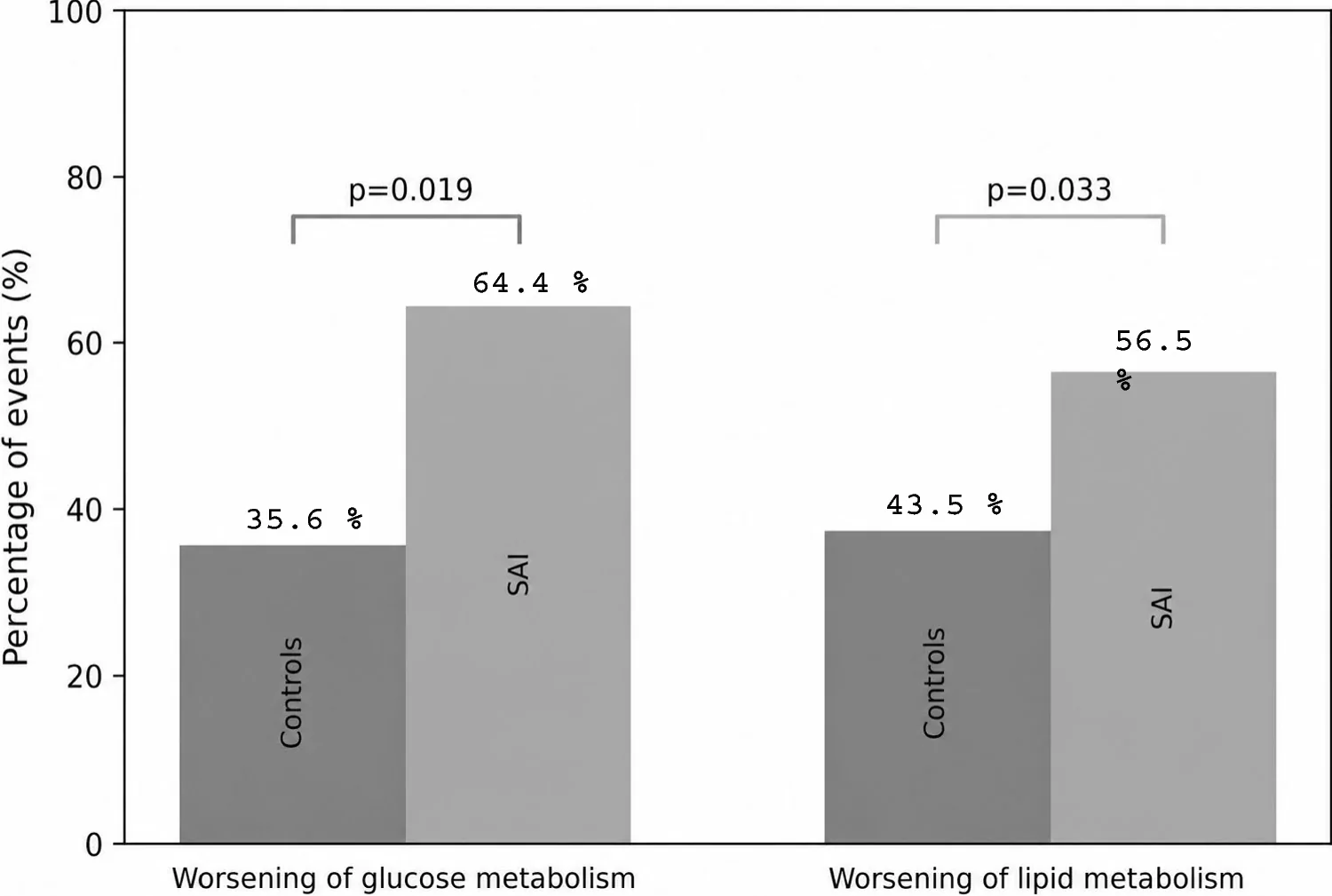
Incidence of worsening of glucose and lipid metabolism in patients affected by SAI and on GCs replacement therapy and in controls. Histogram. Univariate analysis

After 3 years of follow-up, lipid profile worsened in 92 patients (65.7%). Patients suffering from SAI and therefore treated with GC replacement therapy had a higher incidence of dyslipidemia (56.5% *p* = 0.033, OR: 2.16, 95% IC 1.1–4.4) than controls (43.5%, OR: 0.66, 95% IC: 0.44–0.99) (Fig. 1 and Table 2). Among the whole study population, the worsening of lipid profile occurred more frequently in younger individuals (45 years IQR: 26) compared to those whose lipid levels remained normal or unchanged (60 years IQR: 35 *p* = 0.004). The worsening of lipid metabolism occurred more frequently in patients with hypogonadal hypogonadism (78.7%, *p* = 0.021), in patients with TSH deficit (80%, *p* = 0.008), in patients with IGT/T2DM at baseline (91.7%, *p* = 0.05), and in patients with dyslipidemia at baseline (100%, *p* = 0.001), as detailed in Table 4.

**Table 4:** Lipid metabolism outcome at 3 years of GCs replacement therapy in patients with SAI and at 3 years of observation in controls, according to different clinical and biochemical features. Univariate analysis

		Dyslipidemia at FUP		
		Normal/unchanged	Worsening	*p*-value
Number of patients		48 (34.3%)	92 (65.7%)	
Gender				0.189
Males n, (%)		21 (29.2%)	51 (70.8%)	
Females n, (%)		27 (39.7%)	41 (60.3%)	
Age median years (IQR)		60 (35)	45 (26)	0.004
Primary pituitary diseases				0.354
Not-secreting lesions n, (%)		24 (29.6%)	57 (70.4%)	
Cushing disease n, (%)		1 (16.7%)	5 (83.3%)	
Acromegaly n, (%)		14 (40%)	21 (60%)	
Hyperprolactinemia n, (%)		9 (50%)	9 (50%)	
Hypogonadal hypogonadism				0.021
Yes, n (%)		10 (21.3%)	37 (78.7%)	
No, n (%)		38 (40.9%)	55 (59.1%)	
Therapy for hypogonadal hypogonadism				0.022
Yes, n (%)		5 (16.7%)	25 (83.3%)	
No, n (%)		43 (39.1%)	67 (60.9%)	
Secondary TSH deficit				0.008
Yes, n (%)		10 (20%)	40 (80%)	
No, n (%)		38 (42.2%)	52 (57.8%)	
Levothyroxine replacement therapy				0.07
Yes, n (%)		21 (27.6%)	55 (72.4%)	
No, n (%)		27 (42.2%)	37 (57.8%)	
GHD				0.196
Yes, n (%)		7 (24.1%)	22 (75.9%)	
No, n (%)		41 (36.9%)	70 (63–1%)	
Hormone replacement therapy with rhGH				0.635
Yes, n (%)		4 (28.6%)	10 (71.4%)	
No, n (%)		44 (34.9%)	82 (65.1%)	
AVP deficit				0.224
Yes, n (%)		3 (21.4%)	11 (78.6%)	
No, n (%)		45 (35.7%)	81 (64.3%)	
Glucocorticoid type				0.756
Hydrocortisone n, (%)		12 (25%)	36 (75%)	
Cortisone Acetate n, (%)		6 (28.6%)	15 (71.4%)	
Hydrocortisone or equivalent dose mg/day median (IQR)		30 (25)	20 (20)	0.076
HCEq/BSA		14.6 (11.4)	10.6 (8.9)	0.032
Hydrocortisone dose or equivalent	< 20 mg daily	3 (16.7%)	22 (42.3%)	0.128
	20–29 mg daily	3 (16.7%)	9 (17.3%)	
	> 30 mg daily	12 (66.7%)	21 (40.4%)	
Glucose metabolism at baseline				0.05
Normal n, (%)		47 (36.7%)	81 (63.3%)	
IGT/DM2 n, (%)		1 (8.3%)	11 (91.7%)	
HDL- cholesterol (mg/dL)		51 (25)	52.5 (28)	0.389
LDL- cholesterol (mg/dL)		137 (30)	117 (58)	0.597
Triglycerides (mg/dL)		80 (105)	121 (85)	0.06
Dyslipidemia at baseline				0.001
No n, (%)		48 (41.7%)	67 (58.3%)	
Yes n, (%)		0 (0%)	25 (100%)	
Lipid metabolism treatment				0.001
None n, (%)		37 (88.1%)	5 (11.9%)	
Diet n, (%)		4 (33.3%)	8 (66.7%)	
Ezetimibe, n (%)		7 (18.9%)	30 (81.1%)	
Fenofibrate, n (%)		0 (0%)	14 (100%)	
Statin, n (%)		0 (0%)	35 (100%)	

#### Logistic regression

**Fig. 2:**
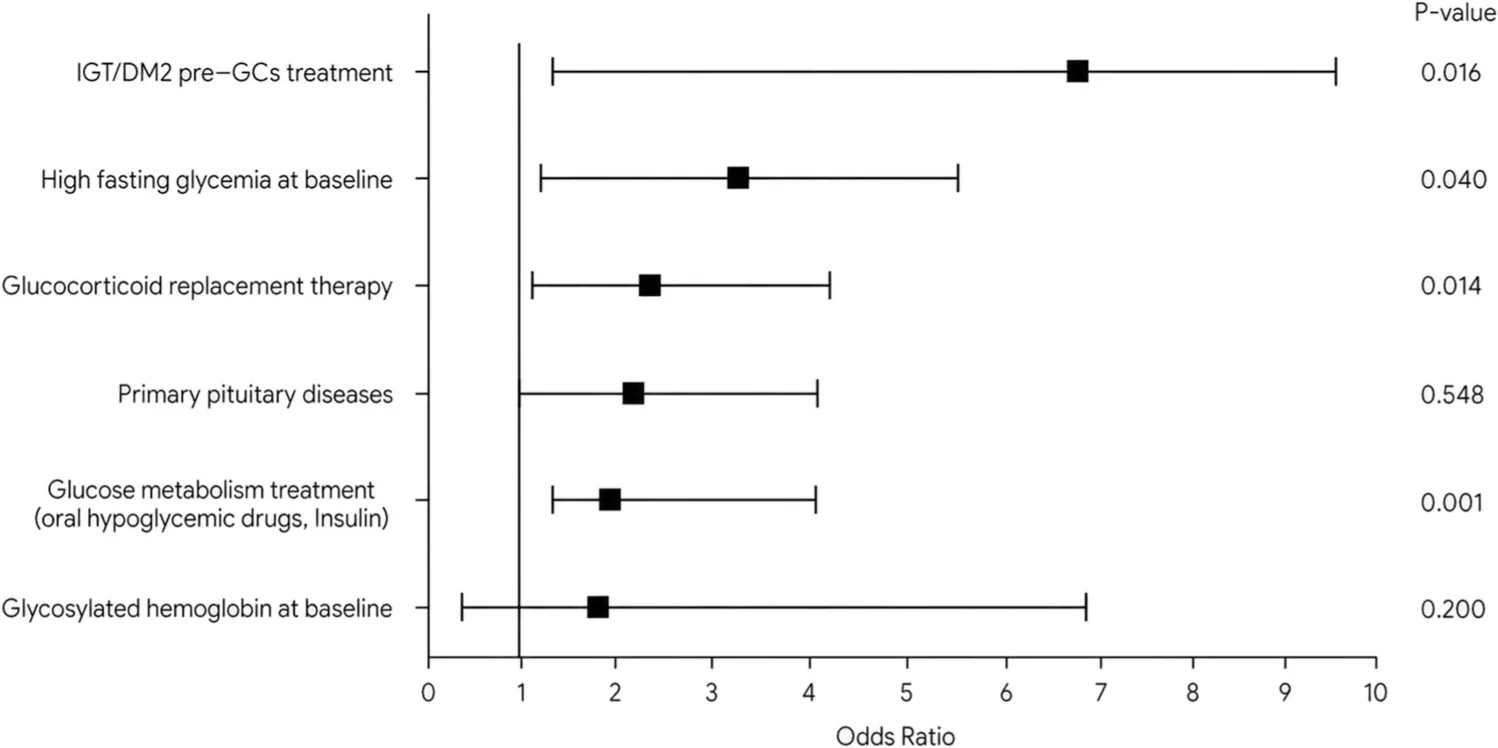
Factors associated with worsening of glucose metabolism. Results of logistic regression are represented as odds ratios (ORs) in forest plot

**Fig. 3:**
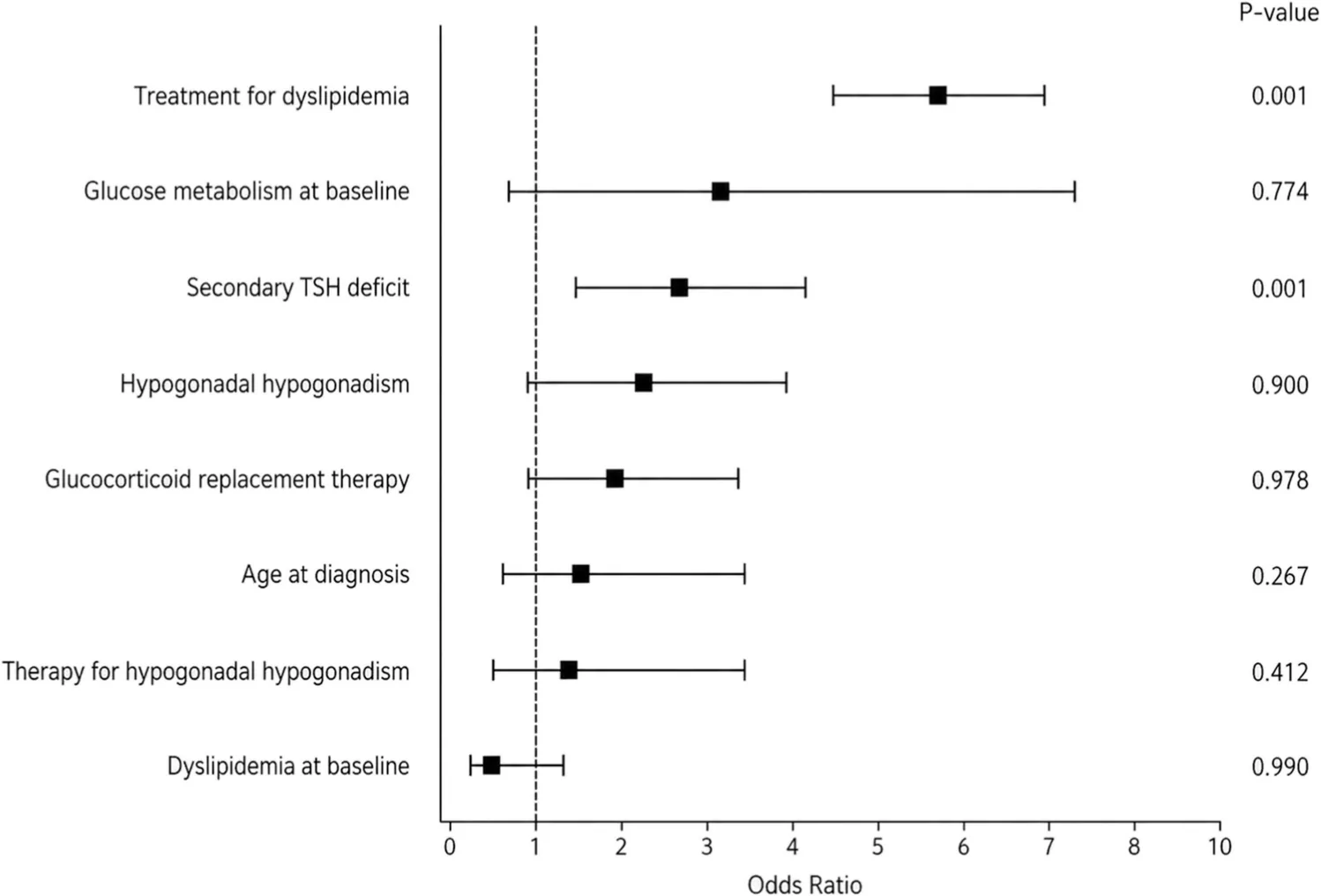
Factors associated with worsening of lipid metabolism. Results of logistic regression are represented as odds ratios (ORs) in forest plot

GC replacement therapy (*p* = 0.014, OR: 2.3, 95%IC: 1.1–4.9), higher fasting glycaemia at baseline (*p* = 0.04, OR: 7.6, 95%IC: 2.3–25.4) and IGT/T2DM at baseline (*p* = 0.016, OR: 8.6, 95%IC: 0.8–57) were the main risk factors for the worsening of glucose metabolism at 3-year follow-up, as shown in Fig. 2. TSH deficit and treatment for dyslipidemia were the main risk factors for the worsening of lipid profile (respectively *p* < 0.001, OR: 2.9, 95%IC: 1.3–6.5; and *p* < 0.001 OR:8.2, 95%IC: 4.5–15) (Fig. 3).

## Discussion

The treatment of patients with SAI is still a clinical challenge to identify the most appropriate type of GCs and dose of replacement therapy, to simulate the physiological circadian rhythm of cortisol secretion. Several studies have proved that patients with SAI on replacement therapy have an increased prevalence of cardiovascular risk and adverse metabolic profiles, such as abdominal obesity, elevated blood pressure, dyslipidemia [28], and IGT or IFG [29, 30]. Exposure to excessive doses of exogenous GCs plays a key role in altering the glucometabolic profile and increasing cardiovascular risk [12]. In this study, we investigated the changes of glucose and lipid metabolism after 3 consecutive years of treatment with substitutive doses of GCs for SAI, in comparison with a control group of patients affected by pituitary diseases but without SAI. Our results provided evidence that patients with SAI on GC replacement therapy experienced a worsening of glucose homeostasis in 64.4% of cases and a worsening of lipid homeostasis in 56.5% of cases, significantly more frequently than as observed in controls. The results of this study are consistent with some previous data [18, 29]. Although GC replacement therapy has been associated with an increased risk of cardiovascular disease and mortality [12], actual evidence remains not conclusive. Stewart et al. conducted a retrospective, observational study, demonstrating that treatment with hydrocortisone at a daily dose of less than 50 mg/day for 6–12 months was associated with an increased risk of T2DM, dyslipidemia and arterial hypertension, compared to healthy controls [18]. Furthermore, Rahvar et al. reported that patients on conventional hydrocortisone replacement therapy (at a mean dose of 27 mg/day) had higher blood glucose levels than healthy controls [31]. Further studies in literature instead did not report a correlation between GCs replacement therapy and changes in fasting blood glucose, total cholesterol, cLDL, HDL-cholesterol and triglycerides at 12 months of treatment nor in glucose after OGTT and insulin sensitivity [19, 32]. Data from the literature on lipid metabolism showed that patients receiving higher doses of GCs replacement therapy experience a noticeable increase in total cholesterol, adiponectin, cLDL and triglycerides, and a decrease in HDL [33, 34].

Interestingly, our results showed that GCs replacement therapy is not an independent risk factor for the development of alterations in glucose metabolism. In fact, patients with pre-existing IGT/DM2, with higher fasting glucose and on treatment with lowering glucose therapy and/or insulin (before starting GC replacement therapy) had a significantly increased risk of developing further deterioration of glucose metabolism. This is consistent with robust evidence from guidelines and longitudinal studies that supports the role of IGT as a significant risk factor for developing T2DM [35].

Other findings moreover suggested that the worsening of glucose metabolism may be related to the GC replacement therapy rather than to the condition of hypopituitarism, possibly for the use of hormone replacement therapies that may mitigate the negative effects of untreated hypopituitarism on glucose metabolism.

Furthermore, although our results suggested an association between the worsening of lipid metabolism in patients on GCs replacement therapy, this result was not confirmed in the logistic regression, which reported the TSH deficit as a single independent prognostic factor. We may speculate that, although all patients in our cohort achieved normal levels of fT4 on levothyroxine replacement therapy, the central hypothyroidism “per-se” may impact on lipid homeostasis [36, 37].

Another interesting finding of our study is the lack of a correlation between the daily dose of GC replacement therapy and the changes in glucose and lipid metabolism. In fact, as reported by Steward and coauthors the worsening of glucose metabolism occurs more frequently in patients on treatment with 50 mg or less of daily hydrocortisone [18]. Previous studies in the literature have demonstrated however that a daily dose of 20 mg hydrocortisone is the threshold for distinguishing patients who may or may not develop metabolic complications [19].

This study has several limitations. The retrospective nature of the study and the relatively small sample size may limit the generalizability of our findings and reduce the statistical power to detect smaller differences between groups, possibly underestimating differences between different dosages of GCs replacement therapy. Another study limitation was the high prevalence of males in the group of patients with SAI. Although it can be hypothesized that the gender difference between the two groups may have influenced carbohydrate and metabolic output, this possible effect is not confirmed in the univariate study. Another limitation that needs to be considered is that the doses of GC replacement therapy are quite higher in our cohort with respect to those reported in further studies and potential long-term side effects of GCs replacement therapy may be underestimated in our cohort for the established observation period of three years, according to the study design.

However, the median dose of hydrocortisone or equivalent in our cohort was 25 mg/daily, that is in accordance with those reported by the Endocrine Society guidelines, suggesting a daily glucocorticoid replacement dose of between 15 and 25 mg, with a midpoint of 20 mg of hydrocortisone [38].

Finally, the potential impact of prior hormonal hypersecretion due to the presence of secreting PAs/PitNETs in our cohort must be considered when interpreting the observed alterations in long-term parameters.

In conclusion, our study proved that GCs replacement therapy may be associated with a worsening of glucose metabolism, particularly in patients already affected by IGT/T2DM before starting GCs replacement therapy. Moreover, our results also suggest that these subgroup of patients should be more strictly monitored, to early identify alterations of glucose metabolism. GCs hormone replacement therapy should be tailored in a holistic view, taking into account the complex clinical scenarios of patients with other concomitant metabolic disorders and multiple pituitary hormone deficits.

## Data Availability

The data sets generated during and/or analyzed during the current study are not publicly available, but are available from the corresponding author on reasonable request.
